# Association between body mass index and newly diagnosed drug-resistant pulmonary tuberculosis in Shandong, China from 2004 to 2019

**DOI:** 10.1186/s12890-021-01774-2

**Published:** 2021-12-06

**Authors:** Wan-mei Song, Jing Guo, Ting-ting Xu, Shi-jin Li, Jin-yue Liu, Ning-ning Tao, Yao Liu, Qian-yun Zhang, Si-qi Liu, Qi-qi An, Yi-fan Li, Chun-bao Yu, Ji-hua Dong, Huai-chen Li

**Affiliations:** 1 Department of Respiratory and Critical Care Medicine, Shandong Provincial Hospital Affiliated to Shandong University, Shandong Provincial Hospital Affiliated to Shandong First Medical University, Jinan, 250021 Shandong People’s Republic of China; 2 Cheeloo College of Medicine, Shandong University, Jinan, 250012 Shandong People’s Republic of China; 3 School of Medicine and Life Sciences, Shandong First Medical University and Shandong Academy of Medical Sciences, Taian, 271016 Shandong People’s Republic of China; 4 Department of Emergency, The Fifth People’s Hospital of Jinan, Jinan, 250031 Shandong People’s Republic of China; 5 Department of Intensive Care Unit, Shandong Provincial Third Hospital, Jinan, 100191 Shandong People’s Republic of China; 6 Department of Respiratory and Critical Care Medicine, Beijing Hospital, Beijing, 100730 People’s Republic of China; 7 Graduate School of Peking Union Medical College, Chinese Academy of Medical Sciences and Peking Union Medical College, Beijing, 100730 People’s Republic of China; 8 Katharine Hsu International Research Center of Human Infectious Diseases, Shandong Provincial Chest Hospital, Jinan, 250013 Shandong People’s Republic of China; 9 Department of Respiratory Medicine, Heze Mudan People’s Hospital, Heze, 274000 Shandong People’s Republic of China; 10 College of Traditional Chinese Medicine, Shandong University of Traditional Chinese Medicine, Jinan, 250355 Shandong People’s Republic of China

**Keywords:** Tuberculosis, Drug resistance, Body mass index, Overweight, Underweight

## Abstract

**Background:**

Drug-resistant tuberculosis (DR-TB), obesity, and malnutrition are growing public health problems in the world. However, little has discussed the impact of different BMI status on the emergence of TB drug resistance. We aimed to explore the drug-resistant profiles of DR-TB and its clinical predictors among underweight, overweight or obesity population.

**Methods:**

8957 newly diagnosed TB cases with drug susceptibility results and BMI data in Shandong China, from 2004 to 2019 were enrolled. Multivariable and univariable logistic regression models were applied to investigate the impact of BMI on different drug-resistance. Clinical predicators and drug-resistant profiles of DR-TB among obesity, underweight, normal TB group were also described.

**Results:**

Among 8957 TB cases, 6417 (71.64%) were normal weight, 2121 (23.68%) were underweight, 373 (4.16%) were overweight, and 46 (0.51%) were obese. The proportion of drug resistance and co-morbidity among normal weight, underweight, overweight, obese TB groups were 18.86%/18.25%/20.38%/23.91% (DR-TB), 11.19%/11.74%/9.65%/17.39% (mono-resistant tuberculosis, MR-TB), 3.41%/3.06%/5.36%/0.00% (multidrug resistant tuberculosis, MDR-TB), 4.21%/3.39%/5.36%/6.52% (polydrug resistant tuberculosis, PDR-TB), 10.57%/8.44%/19.57%/23.91% (co-morbidity), respectively. Compared with normal weight group, underweight were associated with lower risk of streptomycin-related resistance (OR 0.844, 95% CI 0.726–0.982), but contributed to a higher risk of MR-TB (isoniazid) (odds ratio (OR) 1.347, 95% CI 1.049–1.730; adjusted OR (aOR) 1.31, 95% CI 1.017–1.686), P < 0.05. In addition, overweight were positively associated with MDR-TB (OR 1.603, 95% CI 1.002–2.566; aOR 1.639, 95% CI 1.02–2.633), isoniazid + rifampicin + streptomycin resistance (OR 1.948, 95% confidence interval (CI): 1.061–3.577; aOR 2.113, 95% CI 1.141–3.912), Any isoniazid + streptomycin resistance (OR 1.472, 95% CI 1.013–2.14; aOR 1.483, 95% CI 1.017–2.164), *P* < 0.05.

**Conclusions:**

The higher risk of MDR-TB, isoniazid + rifampicin + streptomycin resistance, Any isoniazid + streptomycin resistance, and co-morbidity among overweight population implies that routine screening for drug sensitivity and more attention on co-morbidity among overweight TB cases may be necessary. In addition, underweight TB cases have a higher risk of isoniazid resistance. Our study suggests that an in-depth study of the interaction between host metabolic activity and infection of DR-TB may contribute more to novel treatment options or preventive measures, and accelerate the implementation of the STOP TB strategy.

## Background

Tuberculosis (TB), is caused by *Mycobacterium tuberculosis* (MTB), contributes to a largest number of deaths from infectious diseases (more than HIV/AIDS) [1, 2]. According to WHO Tuberculosis Report, approximately 10.0 million population suffered from TB globally in 2018, and the prevalence was 132 cases per 100,000 people [1]. Additionally, nearly 1.2 million HIV-negative and 251, 000 HIV-positive patients died from TB in 2018 [1]. Nine percent of the overall TB burden worldwide were from China, only second to India (27%) [1].

Multidrug-resistant tuberculosis (MDR-TB) is defined as TB with resistance to at least isoniazid (INH) and rifampin (RFP), which is usually associated with longer hospitalization, more expensive treatment, and higher mortality [3]. The bacteriological cause is the transmission of MDR-TB strains in new cases or the acquired resistance through de novo mutation during TB treatment [2, 3]. The emergence and spread of rifampin-resistant (RR-TB) or MDR-TB has brought grim challenge to TB control, and threatens the health of human being. The estimated proportions of RR-TB/MDR-TB among previously treated and newly diagnosed cases were 18% (95% confidence interval [CI] 7.6–31%) and 3.4% (95% CI 2.5–4.4%) respectively [1]. Despite recent advances in early diagnosis of MDR-TB such as GeneXpert MTB/RIF, laboratories in many low-income countries are still unable to carry out routine bacterial culture and drug susceptibility testing (DST), thus clinical predictors of DR-TB especially MDR-TB may contribute to the early discovery and timely treatment of suspected cases [4, 5]. The identification of clinical risk factors for the development of drug-resistant tuberculosis (DR-TB) will also benefit the management of TB patients and facilitate tuberculosis control programs [1, 5].

The critical influences of diverse nutritional status on TB infection have been recognized for decades [6]. Recent years, there were more and more researches focused on body mass index (BMI, usually used as one of the indicators to judge nutritional status) and TB, for example, the impact of BMI on TB incidence, TB treatment outcomes, TB-related mortality, or negative conversion rate of new sputum among MDR-TB patients [7–9]. Lower BMI is a recognized risk factor for active tuberculosis infection [10]. The main causes of malnutrition were poverty and food shortages [7, 8]. Malnutrition could damage the immunity of human body by decreasing the concentration of T-cell subset (including T-cytotoxic-suppressor cell, T helper cells, and natural killer cells), immunoglobulins, and interleukin-2 receptor, making them more vulnerable to infections such as TB and HIV [11]. Although, former researches revealed that the increased incidence of different infections such as hospital-acquired or postoperative infections might be attributable to obesity [12], available evidence on obesity and risk of active tuberculosis infection indicated an inverse relationship [6]. Interestingly, a study in rural China found that BMI more than 28.0 kg/m^2^ was independent risk factor of latent tuberculosis infection (LTBI, adjusted odds ratio (aOR) 1.17, 95% CI 1.04–1.33) [13]. In summary, BMI have an influence on the infection of MTB strains, and it may also affect the infection of resistant strains. However, no previous publications seem to have examined the impact of different BMI levels on primary drug resistance among TB cases [7–9].

This research intended to explore the association between BMI and primary DR-TB in the aspects as follows: (1) to describe the clinical characteristics of TB cases with four different BMI status (underweight/normal weight/overweight/obese); (2) to illustrate the drug resistant profiles of TB cases subgroups stratified by BMI; (3) to analyze the relative risk of DR-TB including DR-TB (total), MDR-TB (total), mono-resistant tuberculosis (MR-TB, total), polydrug resistant tuberculosis (PDR-TB, total), RFP-related resistance, INH-related resistance, streptomycin (SM)-related resistance, MR-TB (INH), INH + RFP + SM resistance (MDR3), INH + SM resistance (PDR2), Any INH + SM resistance among subgroups with different BMI; (4) to investigate the risk factors of DR-TB among TB cases subgroups stratified by BMI.

## Methods

### Statement

The Ethics Committee of Shandong Provincial Hospital (SPH) and Shandong Provincial Chest Hospital (SPCH) approved for our study. Personal information of TB patients such as names were erased before data analysis and reporting. All methods were performed in accordance with relevant guidelines and regulations. Informed consent was obtained from all participants or, if participants are under 18, from a parent and/or legal guardian.

### Setting

Our study was conducted in Shandong, a coastal province in eastern China (36°24′N latitude and 118° 24′ E longitude), with 100 million inhabitants and an area of 157,100 km^2^ [14]. A large-scale nationwide survey found that 211,900 population in Shandong suffered from TB in 2010 [15], and its incidence had reduced from 40.8 to 26.25 per 100,000 from 2005 to 2017 [16]. The rates of MR-TB, MDR-TB, PDR-TB among newly diagnosed TB cases in Shandong during 2018 were 13.35%, 3.73%, 4.30%, respectively [17]. As estimated, the overall standardized prevalence of combined overweight and obesity among Chinese adults (≥ 40 years) were 30.3% (95% CI 30.1–30.4), and the prevalence of obesity (BMI ≥ 30 kg/m^2^) was 3.5% (95% CI 3.4–3.6) [18].

### Data collection and definitions

**Fig. 1:**
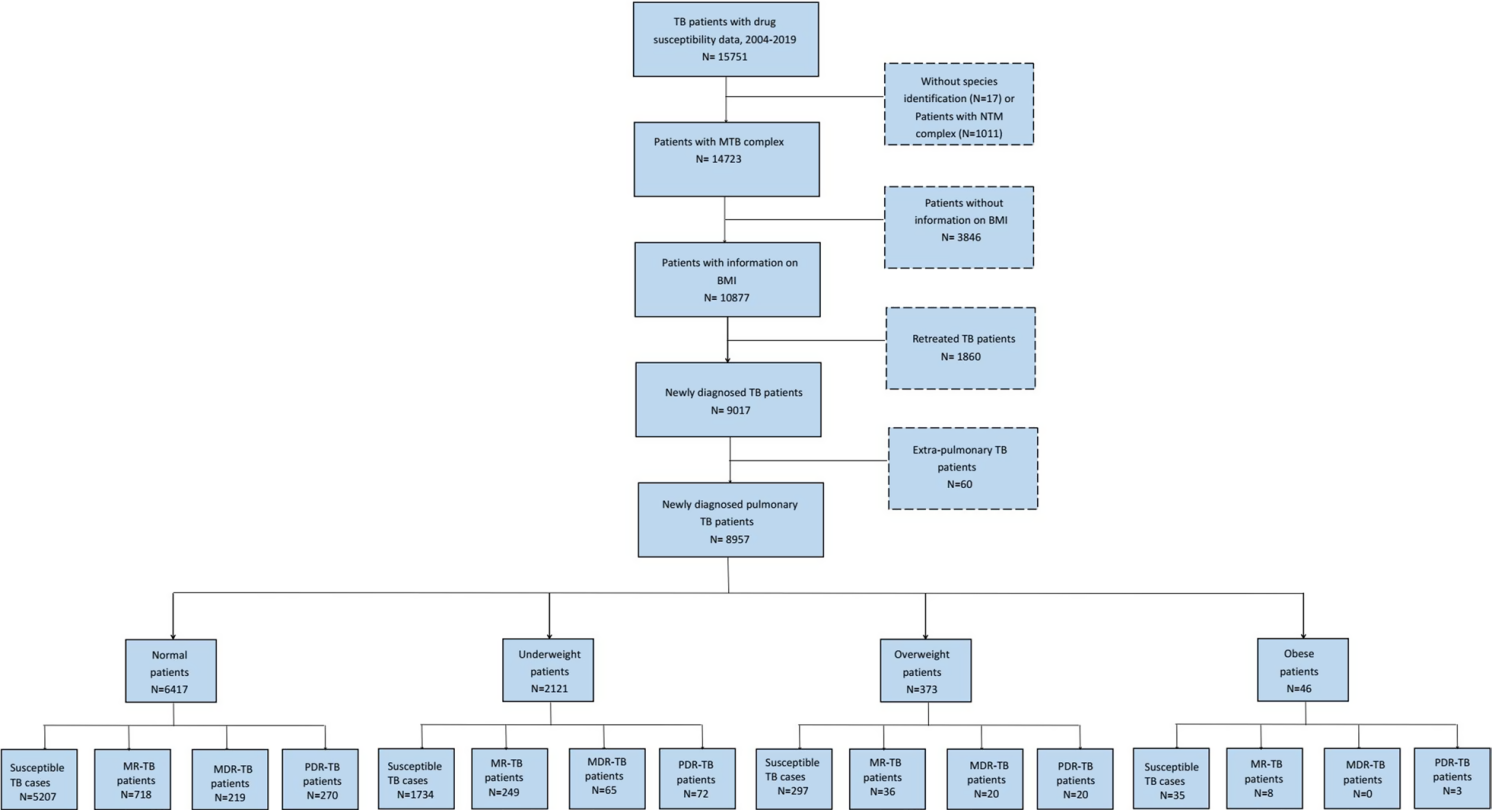
Flow chart of patient inclusion. *Notes* TB, tuberculosis; BMI, body mass index; MTB, *Mycobacterium tuberculosis;* NTM, *Non-tuberculous mycobacterium;* MR-TB, mono-resistant tuberculosis; MDR-TB, multidrug resistant tuberculosis; PDR-TB, polydrug resistant tuberculosis; Underweight: BMI < 18.5 kg/m^2^; Normal weight: 18.5 kg/m^2^ ≥ BMI ≤ 24.9 kg/m^2^; Overweight: 25 kg/m^2^ ≥ BMI ≤ 29.9 kg/m^2^; Obese: BMI ≥ 30 kg/m^2^

8957 newly diagnosed pulmonary TB patients with BMI status and DST results were collected from 34 monitoring sites of DR-TB in Shandong, SPH and SPCH from 1 Jan 2004 to 31 Dec 2019 retrospectively. Only patients with positive sputum smear specimens from 34 monitoring sites in Shandong were enrolled, and then these positive specimens were sent to the laboratory center in SPCH for strain identification and drug susceptibility tests (DST), meanwhile their basic demography and clinical characteristics were collected. Information of BMI, age, sex (male or female), drinking (yes or no), smoking (yes or no), cavity (yes or no), and co-morbidity were collected through questionnaire. Nutritional status indicators except BMI were not routinely collected. Exclusion criteria: (1) cases with previous TB history; (2) *Nontuberculosis mycobacteria* (NTM) infection; (3) BMI information missing; (4) extra-pulmonary cases (Fig. 1). We excluded extra-pulmonary TB patients because there were only 60 patients with extra-pulmonary tuberculosis, and those patients were more complicated than pulmonary TB patients.

BMI below 18.5 kg/m^2^ were in the underweight range, between 18.5 and 24.9 kg/m^2^ were in normal weight range, between 25 and 29.9 kg/m^2^ were in the overweight range, ≥ 30 kg/m^2^ were in the obese range [19]. Mono-resistance (MR) was defined as resistance to only one anti-TB drug of first-line [20]. Multidrug resistance (MDR) refers to resistance to at least both INH and RFP [20]. Polydrug resistance (PDR) was defined as resistance to at least two anti-TB drugs of first-line, but except resistance to both INH and RFP [20]. MR-TB (INH) refers to TB patients who are resistant only to isoniazid and sensitive to other drugs. INH-related resistance was defined as resistance to at least isoniazid.

### Laboratory methods

Bacteriological culture, strain identification, and phenotypic DST were all conducted in Katharine Hsu International Research Center of Human Infectious Diseases (KICID) of SPCH. Initially, at least two sputum specimens of each eligible patient were collected. Then, isolates were cultured in Löwenstein-Jensen (L-J) medium according to the standard protocol, and then these growing colonies were used for strain identification and phenotypic DST [17]. DST for four anti-TB drugs of first-line (INH, 0.2 μg/mL; RFP, 40 μg/mL; SM, 10 μg/mL; ethambutol (EMB), 2 μg/mL were routinely conducted through absolute concentration method on L-J media. Standard traditional biochemical testing such as 16S rRNA gene sequence analysis were applied for strain identification [17]. The above steps were completed independently by at least two professionally trained investigators, and Superior TB National Reference laboratory in SPCH was responsible for external quality assessment.

### Data analysis

Categorical baseline demographic and clinical features of 8957 TB cases including age (0–14, 15–24, 25–44, 45–64, 65 +), sex (men or women), smoker or non-smoker, drinker or non-drinker, cavity (yes/no), co-morbidity (yes/no), and drug resistant profiles in four subgroups with different BMI status were compared by Pearson Chi-square test or Fisher’s exact test. The age subgroups were divided according to previous publications [21, 22]. Binary logistic regression models including univariable and multivariable analysis were applied to investigate the correlation between BMI status and various drug resistant sub-types. Due to the mutually exclusive outcomes of MR, MDR, and PDR, we also carried out univariable and multivariable multinomial logistic regression analysis. Moreover, logistic regression models were also used to estimate the risk factors of primary drug resistance among normal weight, overweight, or obese TB group, respectively. According to published studies, categorical covariates including age, sex, smoking status, alcohol use, cavity (yes/no), co-morbidity were adjusted for multivariable analysis [17]. Statistically significant refers to a two-sided *P* value < 0.05. All data analyses were conducted in SPSS software (version 20.0).

## Results

### Patients’ characteristics

As shown in Table 1, 8957 newly diagnosed pulmonary TB patients with DST results and BMI status were enrolled in our analysis, of which 6417 (71.64%) were normal weight, 2121 (23.68%) were underweight, 373 (4.16%) were overweight, and 46 (0.51%) were obese. Of the overall patients, 15.62% aged between 15 and 24, 25.31% aged between 25 and 44, 33.16% aged between 45 and 64, 25.63% aged more than 65, 83.01% were males, 10.95% have co-morbidity such as diabetes (6.84%), chronic obstructive pulmonary disease (COPD, 1.88%), hypertension (6.84%), HIV/AIDS or other immunocompromised diseases (7/8957, 0.08%).

**Table 1:** Baseline characteristics of 8957 newly diagnosed pulmonary TB patients in Shandong, China, 2004–2019

Characteristics	Total (n = 8957)	BMI category (kg/m^2^)				P value		
		Normal weight TB cases (n = 6417)	Underweight TB cases (n = 2121)	Overweight TB cases (n = 373)	Obese TB cases (n = 46)	Underweight group vs normal weight group	Overweight group vs normal weight group	Obese group vs normal weight group
*Age (years)*								
0–14	25/8920 (0.28%)	12/6392 (0.19%)	11/2110 (0.52%)	1/372 (0.27%)	1/46 (2.17%)	0.011*	0.521	0.089
15–24	1393/8920 (15.62%)	999/6392 (15.63%)	368/2110 (17.44%)	23/372 (6.18%)	3/46 (6.52%)	0.049*	< 0.001***	0.102
25–44	2258/8920 (25.31%)	1725/6392 (26.99%)	393/2110 (18.63%)	124/372 (33.33%)	16/46 (34.78%)	< 0.001***	0.008**	0.236
45–64	2958/8920 (33.16%)	2187/6392 (34.21%)	607/2110 (28.77%)	148/372 (39.78%)	16/46 (34.78%)	< 0.001***	0.028*	0.936
> 65	2286/8920 (25.63%)	1469/6392 (22.98%)	731/2110 (34.64%)	76/372 (20.43%)	10/46 (21.74%)	< 0.001***	0.254	0.842
*Sex*								
Female	1522/8957 (16.99%)	1103/6417 (17.19%)	339/2121 (15.98%)	65/373 (17.47%)	15/46 (32.61%)	0.199	0.906	0.006**
Male	7435/8957 (83.01%)	5314/6417 (82.81%)	1782/2121 (84.02%)	308/373 (82.8%)	31/46 (67.39%)	0.199	0.906	0.006**
*Cavity*								
No	4501/8013 (56.17%)	3205/5689 (56.34%)	1102/1931 (57.07%)	171/349 (49%)	23/44 (52.27%)	0.575	0.007**	0.588
Yes	3512/8013 (43.83%)	2484/5689 (43.66%)	829/1931 (42.93%)	178/349 (51%)	21/44 (47.73%)	0.575	0.007**	0.588
*Smoking*								
No	5400/7224 (74.75%)	3847/5137 (74.89%)	1266/1725 (73.39%)	261/328 (79.57%)	26/34 (76.47%)	0.217	0.057	0.832
Yes	1824/7224 (25.25%)	1290/5137 (25.11%)	459/1725 (26.61%)	67/328 (20.43%)	8/34 (23.53%)	0.217	0.057	0.832
*Drinking*								
No	5663/7174 (78.94%)	4015/5013 (80.09%)	1353/1708 (79.22%)	266/329 (80.85%)	29/34 (85.29%)	0.435	0.738	0.449
Yes	1511/7174 (21.06%)	1088/5013 (21.7%)	355/1708 (20.78%)	63/329 (19.15%)	5/34 (14.71%)	0.435	0.738	0.449
*Co-morbidity*								
Total	941/8957 (10.95%)	678/6417 (10.57%)	179/2121 (8.44%)	73/373 (19.57%)	11/46 (23.91%)	0.005**	< 0.001***	0.003**
Asthma	44/8957 (0.51%)	24/6417 (0.37%)	18/2121 (0.85%)	2/373 (0.54%)	0/46 (0.00%)	0.007**	0.652	1.000
COPD	181/8957 (2.11%)	114/6417 (1.78%)	54/2121 (2.55%)	12/373 (3.22%)	1/46 (2.17%)	0.027**	0.045*	0.563
Bronchiectasis	35/8957 (0.41%)	24/6417 (0.37%)	11/2121 (0.52%)	0/373 (0.00%)	0/46 (0%)	0.366	0.640	1.000
Silicosis	27/8957 (0.31%)	24/6417 (0.37%)	3/2121 (0.14%)	0/373 (0.00%)	0/46 (0.00%)	0.119	0.640	1.000
Diabetes	588/8957 (6.84%)	451/6417 (7.03%)	76/2121 (3.58%)	52/373 (13.94%)	9/46 (19.57%)	< 0.001***	< 0.001***	< 0.001***
Hypertension	162/8957 (1.88%)	117/6417 (1.82%)	29/2121 (1.37%)	14/373 (3.75%)	2/46 (4.35%)	0.160	0.017*	0.207
Cancer	19/8957 (0.22%)	10/6417 (0.16%)	6/2121 (0.28%)	3/373 (0.8%)	0/46 (0%)	0.241	0.031*	1.000
HIV/AIDS or other immunocompromised diseases	7/8957 (0.08%)	5/6417 (0.08%)	2/2121 (0.09%)	0/373 (0%)	0/46 (0%)	0.687	1.000	1.000

TB, tuberculosis; BMI, body mass index; COPD, chronic obstructive pulmonary disease; HIV/AIDS, human immunodeficiency virus/acquired immune deficiency syndrome

^*^P < 0.05;**P < 0.01;***P < 0.001;

Underweight: BMI < 18.5 kg/m^2^; Normal weight: 18.5 kg/m^2^ ≥ BMI ≤ 24.9 kg/m^2^; Overweight: 25 kg/m^2^ ≥ BMI ≤ 29.9 kg/m^2^; obese: BMI ≥ 30 kg/m^2^

#### Underweight TB cases

Compared with normal BMI cases, TB cases with lower BMI are more likely to be in 15–24 (17.44% vs 15.63%) or > 65 (34.64% vs 22.98%) age group, and they had a lower rate of co-morbidity (8.44% vs 10.57%), *P* < 0.05. Underweight TB cases had a higher proportion of asthma (0.85% vs 0.37%) and COPD (2.55% vs 1.78%) but with a lower rate of diabetes (3.58% vs 7.03%) than normal group, *P* < 0.05. There were no significant differences in sex, cavity, smoking and drinking between lower and normal BMI cases. Both these two groups have more males, but less drinkers, smokers, and cavity (Table 1).

#### Overweight TB cases

The proportions of 25–44 age group (33.33% vs 15.63%), 45–64 age group (39.78% vs 34.21%), cavity (51% vs 43.66%), co-morbidity (19.57% vs 10.57%), COPD (3.22% vs 1.78%), diabetes (13.94% vs 7.03%), hypertension (3.75% vs 1.82%), and cancer (0.80% vs 0.16%) were higher in overweight TB cases than in normal weight cases, *P* < 0.05. In addition, overweight TB cases are less likely to be in 15–24 age group (6.18% vs 15.63%), P < 0.05. There were no significant differences in 0–14 age group, 65 + age group, sex, smoking and drinking between overweight and normal cases (Table 1).

#### Obese TB cases

The rates of females (32.61% vs 17.19%), co-morbidity (23.91% vs 10.57%), diabetes (19.57% vs 7.03%) were higher in obese TB cases than in normal cases, P < 0.05. There were no significant differences in age, smoking, drinking and cavity between obese and normal cases (Table 1).

### Drug resistant profiles

As shown in Table 2, the proportions of drug resistance among normal weight, underweight, overweight, obese TB groups were 18.86%/18.25%/20.38%/23.91% (DR-TB), 11.19%/11.74%/9.65%/17.39% (MR-TB), 3.41%/3.06%/5.36%/0.00% (MDR-TB), 4.21%/3.39%/5.36%/6.52% (PDR-TB). Underweight group had a lower rate of SM-resistance (11.6% vs 13.45%) but higher rate of MR-TB (INH) (4.34% vs 3.26%) than normal weight group, *P* < 0.05. Moreover, there were more MDR-TB (5.36% vs 3.41%) or MDR3 (INH + RFP + SM) (3.22% vs 1.68%) cases among overweight group than normal group, *P* < 0.05. There were no significant differences in other drug resistant sub-types between abnormal weight groups and normal group.

**Table 2:** Drug-resistant profiles among newly diagnosed pulmonary TB patients with different BMI

Drug resistance	Normal weight DR-TB cases (1210/6417, 18.86%)	Underweight DR-TB cases (387/2121, 18.25%)	Overweight DR-TB cases (76/373, 20.38%)	Obese DR-TB cases (11/46, 23.91%)	P value		
					Underweight group vs Normal weight group	Overweight group vs Normal weight group	Obese group vs Normal weight group
*DR-TB*	1210 (18.86%)	387 (18.25%)	76 (20.38%)	11 (23.91%)	0.532	0.467	0.383
*Any resistance to first-line drug*							
INH	663 (10.33%)	218 (10.28%)	47 (12.6%)	5 (10.87%)	0.944	0.164	0.905
RFP	311 (4.85%)	94 (4.43%)	23 (6.17%)	0 (0.00%)	0.436	0.252	0.171
EMB	95 (1.48%)	31 (1.46%)	6 (1.61%)	0 (0.00%)	0.950	0.842	1.000
SM	863 (13.45%)	246 (11.6%)	60 (16.09%)	9 (19.57%)	0.028*	0.149	0.226
*MR-TB (Total)*	718 (11.19%)	249 (11.74%)	36 (9.65%)	8 (17.39%)	0.488	0.358	0.184
INH	209 (3.26%)	92 (4.34%)	9 (2.41%)	2 (4.35%)	0.019*	0.369	0.662
RFP	58 (0.9%)	18 (0.85%)	1 (0.27%)	0 (0.00%)	0.815	0.379	1.000
EMB	12 (0.19%)	5 (0.24%)	1 (0.27%)	0 (0.00%)	0.662	0.521	1.000
SM	436 (6.79%)	130 (6.13%)	25 (6.7%)	6 (13.04%)	0.286	0.945	0.094
Others	3 (0.05%)	4 (0.19%)	0 (0.00%)	0 (0.00%)	0.069	1.000	1.000
*MDR-TB (Total)*	219 (3.41%)	65 (3.06%)	20 (5.36%)	0 (0.00%)	0.438	0.047*	0.407
MDR1: INH + RFP	48 (0.75%)	15 (0.71%)	4 (1.07%)	0 (0.00%)	0.849	0.531	1.000
MDR2: INH + RFP + EMB + SM	48 (0.75%)	16 (0.75%)	4 (1.07%)	0 (0.00%)	0.977	0.531	1.000
MDR3: INH + RFP + SM	108 (1.68%)	30 (1.41%)	12 (3.22%)	0 (0.00%)	0.395	0.029*	1.000
Others	15 (0.23%)	4 (0.19%)	0 (0.00%)	0 (0.00%)	1.000	1.000	1.000
*PDR-TB*	270 (4.21%)	72 (3.39%)	20 (5.36%)	3 (6.52%)	0.098	0.284	0.444
PDR1: INH + EMB	6 (0.09%)	2 (0.09%)	1 (0.27%)	0 (0%)	1.000	0.327	1.000
PDR2: INH + SM	219 (3.41%)	55 (2.59%)	17 (4.56%)	3 (6.52%)	0.063	0.241	0.209
PDR3: RFP + SM	27 (0.42%)	8 (0.38%)	2 (0.54%)	0 (0.00%)	0.785	0.672	1.000
PDR4: INH + EMB + SM	10 (0.16%)	3 (0.14%)	0 (0.00%)	0 (0.00%)	1.000	1.000	1.000
Others	8 (0.12%)	4 (0.19%)	0 (0.00%)	0 (0.00%)	0.507	1.000	1.000

TB, tuberculosis; DR-TB, drug-resistant tuberculosis; MR-TB, mono-resistant tuberculosis; MDR-TB, multidrug resistant tuberculosis; PDR-TB, polydrug resistant tuberculosis; EMB, ethambutol; INH, isoniazid; RFP, rifampin; SM, streptomycin; BMI, body mass index;*P < 0.05

Underweight: BMI < 18.5 kg/m^2^; Normal weight: 18.5 kg/m^2^ ≥ BMI ≤ 24.9 kg/m^2^; Overweight: 25 kg/m^2^ ≥ BMI ≤ 29.9 kg/m^2^; Obese: BMI ≥ 30 kg/m^2^

### Association between BMI and primary drug resistance

Table 3 shows the results of univariable and multivariable analysis of the association between BMI and primary anti-tuberculosis resistance. Compared with normal weight group, underweight were associated with lower risk of SM-related resistance (OR 0.844, 95% CI 0.726–0.982, *P* = 0.028), but contributed to a higher risk of MR-TB (INH) (OR 1.347, 95% CI 1.049–1.730, *P* = 0.020; aOR 1.31, 95% CI 1.017–1.686, *P* = 0.037). In addition, overweight was positively associated with MDR-TB (OR 1.603, 95% CI 1.002–2.566, *P* = 0.049; aOR 1.639, 95% CI 1.02–2.633, *P* = 0.041), INH + RFP + SM resistance (OR 1.948, 95% CI 1.061–3.577, *P* = 0.032; aOR 2.113, 95% CI 1.141–3.912, *P* = 0.017), Any INH + SM resistance (OR 1.472, 95% CI 1.013–2.14, *P* = 0.043; aOR 1.483, 95% CI 1.017–2.164, *P* = 0.041). However, there were no statistical differences on drug resistance between obese group and normal weight group. In univariable and multivariable multinomial logistic regression models, we also found that overweight may be a risk factor for MDR-TB (OR 1.601, 95% CI 0.998–2.568, *P* = 0.051; aOR 1.536, 95% CI 0.952–2.476, *P* = 0.078) (see Table 7 in Appendix).

**Table 3:** Association between BMI and primary anti-tuberculosis resistance

The type of drug resistance	Univariable analysis		Multivariable analysis	
	OR (95%CI)	P value	aOR (95%CI)	P value
*Underweight group vs Normal weight group*				
DR-TB	0.96 (0.846–1.09)	0.532	0.977 (0.86–1.111)	0.726
MR-TB	1.041 (0.893–1.215)	0.606	1.056 (0.904–1.233)	0.493
MDR-TB	0.891 (0.672–1.182)	0.424	0.928 (0.698–1.235)	0.610
PDR-TB	0.801 (0.614–1.044)	0.101	0.804 (0.615–1.052)	0.112
INH-related resistance	0.994 (0.846–1.169)	0.944	0.948 (0.746–1.203)	0.659
RFP-related resistance	0.91 (0.719–1.153)	0.436	0.948 (0.746–1.203)	0.659
SM-related resistance	0.844 (0.726–0.982)	0.028*	0.869 (0.746–1.012)	0.071
MR-TB (INH)	1.347 (1.049–1.730)	0.020*	1.31 (1.017–1.686)	0.037*
INH + SM resistance	0.754 (0.558–1.019)	0.066	0.751 (0.554–1.018)	0.065
INH + RFP + SM resistance	0.834 (0.555–1.255)	0.384	0.881 (0.585–1.328)	0.546
Any INH + SM resistance	0.818 (0.656–1.019)	0.073	0.831 (0.665–1.039)	0.105
Susceptible	Reference	Reference	Reference	Reference
*Overweight group vs Normal weight group*				
DR-TB	1.101 (0.849–1.428)	0.467	1.082 (0.832–1.407)	0.559
MR-TB	0.879 (0.617–1.253)	0.476	0.854 (0.596–1.225)	0.391
MDR-TB	1.603 (1.002–2.566)	0.049*	1.639 (1.02–2.633)	0.041*
PDR-TB	1.299 (0.812–2.076)	0.275	1.284 (0.8–2.06)	0.300
INH-related resistance	1.251 (0.912–1.717)	0.165	1.248 (0.907–1.716)	0.174
RFP-related resistance	1.29 (0.833–1.997)	0.253	1.271 (0.817–1.977)	0.287
SM-related resistance	1.234 (0.927–1.641)	0.149	1.224 (0.916–1.636)	0.171
MR-TB (INH)	0.734 (0.374–1.443)	0.371	0.736 (0.373–1.449)	0.375
INH + SM resistance	1.361 (0.82–2.26)	0.234	1.36 (0.815–2.268)	0.239
INH + RFP + SM resistance	1.948 (1.061–3.577)	0.032*	2.113 (1.141–3.912)	0.017*
Any INH + SM resistance	1.472 (1.013–2.14)	0.043*	1.483 (1.017–2.164)	0.041*
Susceptible	Reference	Reference	Reference	Reference
*Obese group vs Normal weight group*				
DR-TB	0.739 (0.374–1.46)	0.384	0.741 (0.374–1.469)	0.391
MR-TB	1.658 (0.766–3.587)	0.199	1.62 (0.747–3.516)	0.222
MDR-TB	–	–	–	–
PDR-TB	1.653 (0.505–5.409)	0.406	1.712 (0.52–5.637)	0.377
INH-related resistance	0.945 (0.372–2.399)	0.905	0.943 (0.37–2.403)	0.901
RFP-related resistance	–	–	–	–
SM-related resistance	0.639 (0.307–1.328)	0.230	0.644 (0.308–1.347)	0.243
MR-TB (INH)	1.350 (0.325–5.607)	0.679	1.304 (0.313–5.435)	0.715
INH + SM resistance	2.038 (0.622–6.678)	0.240	2.089 (0.633–6.892)	0.226
INH + RFP + SM resistance	–	–	–	–
Any INH + SM resistance	1.136 (0.348–3.709)	0.833	1.148 (0.349–3.775)	0.820
Susceptible	Reference	Reference	Reference	Reference

TB, tuberculosis; DR-TB, drug-resistant tuberculosis; MR-TB, mono-resistant tuberculosis; MDR-TB, multidrug resistant tuberculosis; PDR-TB, polydrug resistant tuberculosis; EMB, ethambutol; INH, isoniazid; RFP, rifampin; SM, streptomycin; BMI, body mass index; aOR, adjusted odds ratio; OR, odds ratio;*P < 0.05

Underweight: BMI < 18.5 kg/m^2^; Normal weight: 18.5 kg/m^2^ ≥ BMI ≤ 24.9 kg/m^2^; Overweight: 25 kg/m^2^ ≥ BMI ≤ 29.9 kg/m^2^; Obese: BMI ≥ 30 kg/m^2^

### Risk factors for drug resistance

As shown in Tables 4 and 5, male was a risk factor of DR-TB in underweight (OR 1.385, 95% CI 1.001–1.917, *P* = 0.049; aOR 1.458, 95% CI 1.038–2.048, *P* = 0.030) and overweight cases (OR 2.392, 95% CI 1.044–5.479, P = 0.039; aOR 2.611, 95% CI 1.107–6.158, *P* = 0.028). In addition, co-morbidity except COPD, diabetes, hypertension (aOR 4.34, 95% CI 1.053–17.928, *P* = 0.042) were positively associated with DR-TB in underweight cases. Among normal weight group, male (aOR 1.26, 95% CI 1.059–1.513, P = 0.010) or cavitary (aOR 1.15, 95% CI 1.006–1.319, P = 0.030) pulmonary tuberculosis were more likely to have anti-TB resistance (Table 6). We did not find a significant association between factors including age, smoking, drinking, COPD, diabetes, hypertension and drug resistance among underweight, overweight or normal weight group.

**Table 4:** Univariable and multivariable analysis of risk factors for DR-TB in underweight TB cases

Characteristics	Non-DR n = 1734 (%)	DR-TB n = 387 (%)	Univariable analysis		Multivariable analysis	
			OR (95% CI)	P value	OR (95% CI)	P value
*Age (years)* (n = 1725/n = 385)						
0–14	9 (0.52%)	2 (0.52%)	0.963 (0.203–4.557)	0.962	1.022 (0.213–4.898)	0.978
15–24	299 (17.33%)	69 (17.92%)	Reference	Reference	Reference	Reference
25–44	322 (18.67%)	71 (18.44%)	0.955 (0.662–1.379)	0.808	0.984 (0.68–1.424)	0.932
45–64	479 (27.77%)	128 (33.25%)	1.158 (0.835–1.605)	0.379	1.138 (0.813–1.594)	0.45
> 65	616 (35.71%)	115 (29.87%)	0.809 (0.582–1.124)	0.206	0.789 (0.561–1.109)	0.173
*Sex* (n = 1734/n = 387)						
Female	290 (16.72%)	49 (12.66%)	Reference	Reference	Reference	Reference
Male	1444 (83.28%)	338 (87.34%)	1.385 (1.001–1.917)	0.049*	1.458 (1.038–2.048)	0.030*
*Cavity* (n = 1566/n = 365)						
No	896 (57.22%)	206 (56.44%)	Reference	Reference	Reference	Reference
Yes	670 (42.78%)	159 (43.56%)	1.032 (0.82–1.299)	0.787	1.019 (0.807–1.286)	0.875
*Smoking* (n = 1423/n = 302)						
No	1046 (73.51%)	220 (72.85%)	Reference	Reference	Reference	Reference
Yes	377 (26.49%)	82 (27.15%)	0.77 (0.581–1.019)	0.067	1.157 (0.802–1.669)	0.434
*Drinking* (n = 1407/n = 301)						
No	1108 (78.75%)	245 (81.4%)	Reference	Reference	Reference	Reference
Yes	299 (21.25%)	56 (18.6%)	0.841 (0.639–1.107)	0.216	0.686 (0.455–1.033)	0.071
*Co-morbidity* (n = 1734/n = 387)						
No	1584 (91.35%)	358 (92.51%)	Reference	Reference	Reference	Reference
COPD	47 (2.71%)	7 (1.81%)	0.661 (0.297–1.474)	0.312	0.962 (0.32–2.892)	0.944
Diabetes	63 (3.63%)	13 (3.36%)	0.922 (0.502–1.693)	0.793	0.959 (0.517–1.779)	0.895
Hypertension	23 (1.33%)	6 (1.55%)	1.172 (0.474–2.897)	0.732	1.315 (0.522–3.316)	0.561
Other co-morbidity	97 (5.59%)	15 (3.88%)	0.68 (0.391–1.186)	0.174	0.746 (0.347–1.604)	0.454

TB, tuberculosis; DR-TB, drug-resistant tuberculosis; aOR, adjusted odds ratio; OR, odds ratio; COPD, chronic obstructive pulmonary disease

*P < 0.05; Underweight: BMI < 18.5 kg/m^2^

**Table 5:** Univariable and multivariable analysis of risk factors for DR-TB in overweight TB cases

Characteristics	Non-DR n = 297 (%)	DR-TB n = 76 (%)	Univariable analysis		Multivariable analysis	
			OR (95% CI)	P value	OR (95% CI)	P value
*Age (years)* (*n* = *297/n* = *75*)						
0–14	1 (0.34%)	0 (0%)	NA	NA	NA	NA
15–24	18 (6.06%)	5 (6.67%)	Reference	Reference	Reference	Reference
25–44	99 (33.33%)	25 (33.33%)	0.909 (0.308–2.687)	0.863	0.687 (0.224–2.11)	0.512
45–64	113 (38.05%)	35 (46.67%)	1.115 (0.386–3.221)	0.841	0.84 (0.278–2.534)	0.756
> 65	66 (22.22%)	10 (13.33%)	0.545 (0.165–1.799)	0.319	0.434 (0.121–1.557)	0.2
*Sex* (*n* = *297/n* = *76*)						
Female	58 (19.53%)	7 (9.21%)	Reference	Reference	Reference	Reference
Male	239 (80.47%)	69 (90.79%)	2.392 (1.044–5.479)	0.039*	2.611 (1.107–6.158)	0.028*
*Cavity* (*n* = *281/n* = *68*)						
No	142 (50.53%)	29 (42.65%)	Reference	Reference	Reference	Reference
Yes	139 (49.47%)	39 (57.35%)	1.374 (0.805–2.345)	0.244	1.298 (0.736–2.287)	0.367
*Smoking* (*n* = *259/n* = *69*)						
No	204 (78.76%)	57 (82.61%)	Reference	Reference	Reference	Reference
Yes	55 (21.24%)	12 (17.39%)	1.517 (0.643–3.577)	0.341	0.743 (0.321–1.724)	0.49
*Drinking* (*n* = *260/n* = *69*)						
No	207 (79.62%)	59 (85.51%)	Reference	Reference	Reference	Reference
Yes	53 (20.38%)	10 (14.49%)	0.662 (0.317–1.381)	0.271	0.629 (0.261–1.516)	0.302
*Co-morbidity* (n = 297/n = 76)						
No	239 (80.47%)	59 (77.63%)	Reference	Reference	Reference	Reference
COPD	10 (3.37%)	2 (2.63%)	0.776 (0.166–3.617)	0.746	0.265 (0.031–2.293)	0.228
Diabetes	41 (13.8%)	11 (14.47%)	1.057 (0.515–2.169)	0.881	1.235 (0.563–2.708)	0.599
Hypertension	12 (4.04%)	2 (2.63%)	0.642 (0.141–2.931)	0.567	0.705 (0.138–3.615)	0.676
Other co-morbidity	16 (5.39%)	6 (7.89%)	1.505 (0.568–3.987)	0.411	4.34 (1.053–17.928)	0.042*

TB, tuberculosis; DR-TB, drug-resistant tuberculosis; aOR, adjusted odds ratio; OR, odds ratio; COPD, chronic obstructive pulmonary disease

*P < 0.05; Overweight: 25 kg/m^2^ ≥ BMI ≤ 29.9 kg/m^2^

**Table 6:** Univariable and multivariable analysis of risk factors for DR-TB in normal weight cases

Characteristics	Non-DR n = 5207 (%)	DR-TB n = 1210 (%)	Univariable analysis		Multivariable analysis	
			OR (95% CI)	P value	OR (95% CI)	P value
*Age (years)* (n = 5186/n = 1206)						
0–14	10 (0.19%)	2 (0%)	0.868 (0.189–3.997)	0.856	0.96 (0.209–4.459)	0.963
15–24	812 (15.66%)	187 (0.26%)	Reference	Reference	Reference	Reference
25–44	1356 (26.15%)	369 (0.52%)	1.182 (0.971–1.438)	0.096	1.17 (0.961–1.426)	0.118
45–64	1780 (34.32%)	407 (0.57%)	0.993 (0.819–1.203)	0.942	0.95 (0.781–1.159)	0.622
> 65	1228 (23.68%)	241 (0.34%)	0.852 (0.69–1.052)	0.137	0.829 (0.667–1.03)	0.091
*Sex* (n = 5207/n = 1210)						
Female	918 (17.63%)	185 (15.29%)	Reference	Reference	Reference	Reference
Male	4289 (82.37%)	1025 (84.71%)	1.186 (0.998–1.408)	0.052	1.26 (1.059–1.513)	0.010*
*Cavity* (n = 4624/n = 1065)						
No	2640 (57.09%)	565 (53.05%)	Reference	Reference	Reference	Reference
Yes	1984 (42.91%)	500 (46.95%)	0.86 (0.702–1.054)	0.147	1.15 (1.006–1.319)	0.040*
*Smoking* (n = 4188/n = 949)						
No	3125 (74.62%)	722 (76.08%)	Reference	Reference	Reference	Reference
Yes	1063 (25.38%)	227 (23.92%)	0.924 (0.784–1.09)	0.348	0.984 (0.79–1.226)	0.887
*Drinking* (n = 4160/n = 943)						
No	3257 (78.29%)	758 (80.38%)	Reference	Reference	Reference	Reference
Yes	903 (21.71%)	185 (19.62%)	0.88 (0.738–1.051)	0.158	0.858 (0.68–1.084)	0.2
*Co-morbidity* (n = 5207/n = 1210)						
No	4665 (89.59%)	1074 (88.76%)	Reference	Reference	Reference	Reference
COPD	94 (1.81%)	20 (1.65%)	0.91 (0.562–1.487)	0.718	0.938 (0.5–1.76)	0.843
Diabetes	354 (6.8%)	97 (8.02%)	1.195 (0.946–1.51)	0.136	1.23 (0.969–1.568)	0.089
Hypertension	98 (1.88%)	19 (1.57%)	0.83 (0.507–1.365)	0.466	0.91 (0.548–1.514)	0.72
Other co-morbidity	216 (4.15%)	51 (4.21%)	1.01 (0.744–1.389)	0.917	1.11 (0.746–1.667)	0.596

DR-TB, drug-resistant tuberculosis; aOR, adjusted odds ratio; OR, odds ratio; COPD, chronic obstructive pulmonary disease

*P < 0.05; Normal weight: 18.5 kg/m^2^ ≥ BMI ≤ 24.9 kg/m^2^

## Discussion

Identifying novel risk factors or predictors for DR-TB will facilitate the implementation of TB termination strategies, but so far little studies have explored the relationship between different TB drug-resistance and BMI status. Our study was designed to compare clinical features and drug-resistant profiles of primary pulmonary TB cases with different BMI, thus to provide reference in clinics. We now report DST results and BMI status of 8957 newly diagnosed pulmonary TB patients, and found that abnormal BMI was related to the elevated risk of some drug-resistant sub-types. Meanwhile, BMI were positively associated with the incidence of accompanying diseases among TB cases. Male was a risk factor of DR-TB in underweight, overweight, or normal TB patients.

Obesity has become a global public health problem, and it’s reported that approximately 46% of adults and 15% of children China are overweight or obese [23]. Plenty of evidence have revealed that BMI is negatively associated with active tuberculosis [6, 24]. For instance, a study among 46 028 adult participants in Taiwan found that obesity (BMI ≥ 27 kg/m^2^) (aOR 0.43; 95% CI 0.28–0.67) and overweight (BMI = 24–26.9 kg/m^2^) (aOR 0.67; 95% CI 0.49–0.91) contributed to a lower risk of TB infection [24]. A study in rural China found that BMI ≥ 28.0 kg/m^2^ was observed to be independently associated with LTBI (aOR: 1.17, 95% CI 1.04–1.33) [13]. Interestingly, our study indicated that overweight was a risk factor for MDR-TB, INH + RFP + SM resistance, Any INH + SM resistance. The explanation may be that MTB had a lower virulence due to high-fat exposure and immunoregulation of leptin, and the reproduction speed of MTB with lipid bodies were slower than those without such lipid bodies [25, 26]. Furthermore, MTB can survive without reproducing in fatty tissues, and may go some way towards explaining why higher BMI contributed to LTBI. As we know, primary drug-resistant TB was caused by the transmission of resistant MTB strains, and this resistance may come at a “fitness cost” through a decreased transmission rate, virulence and reproduction speed [27]. Therefore, we supposed that obesity may have a greater impact on reducing the transmission of susceptible MTB strain than some resistant strains, because the transmission of the resistant strains were already at a lower level due to the “fitness cost”, which may explain why there was a higher risk of falling ill with MDR-TB, INH + RFP + SM resistant TB, or Any INH + SM resistant TB among overweight population. Some overweight and obese patients can also be malnourished, since BMI is only one of prognostic factors for assessing malnutrition. Fat accumulation in overweight and obese individuals may induce additional nutritional derangements, both indirectly through acute and chronic diseases with negative impact on nutritional status and directly through metabolic and body composition changes [28]. Furthermore, skeletal muscle mass and function (sarcopenia) may also contribute to malnutrition. Malnutrition in obesity may be an important factor for some increased resistance among newly-diagnosed tuberculosis patient. The indicators for nutritional status include anthropometric index and laboratory parameters [29, 30]. The former mainly includes height, weight, body mass index and alternative indices, trunk measurements (waist and hip circumferences and sagittal abdominal diameter) and limb measurements (mid-upper arm and calf circumferences) and skinfold thickness [29], and the latter mainly includes albumin, prealbumin, urinary creatinine or 3-methylhistidine [30]. However, this retrospective study were not available for other nutritional indexes except BMI. Therefore, it may be a good idea to detect more indicators for nutritional status as above among TB cases to find those with both burden of obesity and malnutrition in future, and then explore their drug-resistant profiles.

Overweight TB cases had a higher possibility of suffering from co-morbidity including COPD, hypertension, diabetes, and cancer. Actually, previous studies have figured out that BMI was a reliable predictor of prevalent diabetes, hypertension, and COPD [31–33]. A study in in South Asian cities among 31,118 participants found that every standard deviation higher of BMI was associated with 1.42 and 1.28 times higher probability of hypertension and 1.65 and 1.60 times higher probability of diabetes among 40–69 years men and women respectively [31]. A dose–response relationship between COPD and BMI has been observed among both males and females, for example the prevalence of COPD increased from 2.5 to 7.6% in men and from 3.5 to 13.4% in women when their BMI changed from normal to obese (BMI ≥ 40) [33]. Recent years, former studies have found that COPD (aOR 1.86, 95% CI 1.01–2.93, P = 0.041) and diabetes (aOR 1.59, 95% CI 1.04–2.44, P = 0.03) could increase the risk of MDR-TB and PDR-TB, respectively [34–36]. Thus, it can be seen that screening for drug resistance among overweight TB cases, especially those combined with COPD or diabetes, may help monitor the development and guide the clinical diagnosis and treatment of DR-TB.

People have found a significant association between underweight and higher risk of active TB, adverse TB treatment outcomes, tuberculosis-related mortality [7, 37–39]. According to a study among the US population in 1971–1975, estimated TB incidence among adults who were underweight, normal, overweight, and obese was 260.2 (95% CI 98.6, 421.8), 24.7 (95% CI 13.0–36.3), 8.9 (95% CI 2.2–15.6), and 5.1 (95% CI 0.0–10.5) per 100,000 person-years, respectively [38]. Interestingly, we found underweight was associated with lower risk of SM-related resistance and co-morbidity, but contributed to a higher risk of MR-TB (INH). Studies have also shown that underweight is associated with abnormal cell-mediated immunity, phagocytic function, complement system, immunoglobulin A secretion, and cytokine production such as reduced secretion of Th1 cytokines (IL-2, TNF-α and interferon-γ), which may lead to more severe TB infection [40]. Moreover, malnourished animals have higher bacterial burdens and impaired immune system (e.g. reduction of reactive N intermediates) [37, 41], Underweight population may influence the infection INH-resistant or SM-resistant MTB strains through immune system, but the molecular mechanism remains to be further explored. Our findings would provide guidance for clinicians when treating underweight TB cases, for instance, they should maintain keen vigilance at INH resistance rather than SM resistance.

It has been observed that males were more likely to be affected with DR-TB among normal, underweight, or overweight population. However, there were inconsistent results in the previous literature about the influence of gender differences on vulnerability to MDR-TB [42–44]. A study conducted in Lianyungang city, China found females were more likely to be infected with MDR-TB (aOR 1.763, 95% CI 1.060–2.934) [42], while Faustini stated that MDR-TB patients tended to be male (OR 1.38; 95% CI 1.16–1.65) in western Europe [43]. We assume that the heterogeneity on the susceptibility of DR-TB among males and females may be related to the their differences in airway structure and lifestyles such as smoking, drinking, stay up late.

This study had some strengths. Firstly, it’s the first study to investigate the effect of BMI on drug resistance among newly diagnosed pulmonary TB cases. Secondly, the relative risk of numerous drug-resistant sub-types including DR-TB (total), MDR-TB (total), MR-TB (total), PDR-TB (total), RFP-related resistance, INH-related resistance, SM-related resistance, IMR-TB (INH), INH + RFP + SM resistance (MDR3), INH + SM resistance (PDR2), Any INH + SM resistance among underweight, overweight, obese were analyzed, and could provide clinical reference for future pathologic and molecular studies on different resistant MTB strains. Thirdly, the large amount (DST results from a province of 100 million people) and long time span (from 2004 to 2019) of our data guaranteed the reliance of our findings.

This study also had some limitations. Firstly, drug resistance of second-line anti-TB drugs were not routinely examined in China unless at the initiative requirements of patients. Thus, the association between BMI and second-line anti-TB resistance remains to be discovered in future. Secondly, this was a retrospective study, and may have information bias. Thirdly, some obese patients can also be malnourished, since BMI is only one of prognostic factors for assessing malnutrition [28, 29, 45]. However, we were not available for other nutritional indexes except BMI due to its retrospective study model. This might be an important confounding factor in the obese group.

## Conclusion

Our study has important implications on the global triple epidemics of underweight, obesity, and DR-TB. The positive effect of overweight on MDR-TB, INH + RFP + SM resistance, Any INH + SM resistance, and co-morbidity implies that routine screening for drug sensitivity and more attention on co-morbidity among overweight TB cases may be necessary. Although patients with normal weight had a lower proportion of drug-resistance than overweight or obese patients in Shandong, screening for drug resistance cannot be ignored either because patients with normal weight accounted for 74.64%. In addition, we found underweight TB cases have a higher risk of INH resistance, which provides a reference for clinical rational use of drugs. Our study suggests that an in-depth study of the interaction between host metabolic activity and infection of DR-TB may contribute more to novel treatment options or preventive measures, and accelerate the implementation of the STOP TB strategy.

## Data Availability

Data can be available through contact with the corresponding author.

## Appendix

See Table 7.

**Table 7:** The associations between TB drug-resistance and BMI through univariable and multivariable multinomial logistic regression analysis

Variable	MR-TB vs SUS-TB				MDR-TB vs SUS-TB				PDR-TB vs SUS-TB			
	Univariable analysis		Multivariable analysis		Univariable analysis		Multivariable analysis		Univariable analysis		Multivariable analysis	
	OR (95% CI)	P value	OR (95% CI)	P value	OR (95% CI)	P value	OR (95% CI)	P value	OR (95% CI)	P value	OR (95% CI)	P value
Normal weight (n = 6417)	Reference	Reference	Reference	Reference	Reference	Reference	Reference	Reference	Reference	Reference	Reference	Reference
Underweight (n = 2121)	1.041 (0.893–1.215)	0.606	1.058 (0.906–1.237)	0.475	0.891 (0.672–1.182)	0.424	0.948 (0.712–1.261)	0.711	0.801 (0.614–1.044)	0.101	0.86 (0.657–1.125)	0.271
Overweight (n = 373)	0.879 (0.617–1.253)	0.476	0.874 (0.612–1.248)	0.458	1.601 (0.998–2.568)	0.051	1.536 (0.952–2.476)	0.078	1.299 (0.812–2.076)	0.275	1.244 (0.775–1.996)	0.366
Obese (n = 46)	1.658 (0.766–3.587)	0.199	1.664 (0.767–3.614)	0.198	0.0000003084 (0-.c)	0.992	0.0000003129 (0-.c)	0.992	1.653 (0.505–5.409)	0.406	1.583 (0.48–5.225)	0.451

TB, tuberculosis; MR-TB, mono-resistant tuberculosis; MDR-TB, multidrug resistant tuberculosis; PDR-TB, polydrug resistant tuberculosis; BMI, body mass index; Underweight: BMI < 18.5 kg/m^2^; Normal weight: 18.5 kg/m^2^ ≥ BMI ≤ 24.9 kg/m^2^; Overweight: 25 kg/m^2^ ≥ BMI ≤ 29.9 kg/m^2^; Obese: BMI ≥ 30 kg/m^2^

## Abbreviations

TB: Tuberculosis
DR-TB: Drug-resistant tuberculosis
WHO: World Health Organization
COPD: Chronic obstructive pulmonary disease
RR-TB: Rifampin-resistant tuberculosis
MDR-TB: Multidrug resistant tuberculosis
MR-TB: Mono-resistant tuberculosis
PDR-TB: Polydrug resistant tuberculosis
LTBI: Latent tuberculosis infection
aOR: Adjusted odds ratio
OR: Odds ratio
CI: Confidence interval
INH: Isoniazide
RFP: Rifampin
EMB: Ethambutol
SM: Streptomycin
MTB: *Mycobacterium tuberculosis*
DST: Drug susceptibility test
NTM: *Non-tuberculous mycobacterium*
HIV/AIDS: Human immunodeficiency virus/acquired immune deficiency syndrome
